# Case Report: Long-term disease control with immunotherapy in primary mandibular adenoid cystic carcinoma presenting with pulmonary metastasis

**DOI:** 10.3389/fimmu.2026.1811044

**Published:** 2026-06-29

**Authors:** Hongcheng Yang, Yanzhen Lai, Qingqing Li, Haijin Yang, Meichen Ji, Xuqiang Luo, Xie Zhu, Yun Li, Huiyu Wu, Peixin Tan, Kunpeng Wu

**Affiliations:** 1 Department of Oncology, Heyuan People's Hospital, Guangdong Provincial People’s Hospital Heyuan Hospital, Heyuan, Guangdong, China; 2 Heyuan Key Laboratory of Molecular Diagnosis & Disease Prevention and Treatment, Doctors Station of Guangdong Province, Heyuan People’s Hospital, Heyuan, Guangdong, China; 3 Department of Pathology, Heyuan People’s Hospital, Guangdong Provincial People's Hospital Heyuan Hospital, Heyuan, Guangdong, China; 4 Department of Radiation Oncology, Guangdong Provincial People’s Hospital (Guangdong Academy of Medical Sciences), Southern Medical University, Guangzhou, China

**Keywords:** immunogenic cell death, immunotherapy, oral fluoropyrimidines, primary intraosseous adenoid cystic carcinoma (PIACC), tumor microenvironment

## Abstract

Primary intraosseous adenoid cystic carcinoma (PIACC) of the mandible is an exceptionally rare malignancy. While radical surgery and radiotherapy provide locoregional control, managing distant metastases remains challenging due to the lack of standard systemic therapies and the characteristically “cold” immune microenvironment of adenoid cystic carcinoma (ACC). We report the case of a 62-year-old male diagnosed with PIACC of the mandible presenting with synchronous bilateral pulmonary metastases (Stage IVC). Following radical resection and adjuvant radiotherapy, the patient received first-line maintenance with oral S-1, achieving stable disease for 36 months. Upon pulmonary progression, after a brief and poorly tolerated trial of sintilimab plus S-1 and subsequent docetaxel, therapy was optimized to a metronomic combination of the PD-1 inhibitor sintilimab and oral capecitabine. This immuno-metronomic regimen achieved durable disease stabilization exceeding 47 months with minimal toxicity. Upon discontinuing immunotherapy due to financial constraints, rapid disease progression ensued, underscoring the critical role of the combination. The patient subsequently received salvage anlotinib, achieving brief stabilization, but passed away from severe pneumonia, yielding a total overall survival of 101 months (~8.4 years). This case challenges the paradigm that ACC is intrinsically refractory to immunotherapy. We hypothesize that systemic interventions, potentially including immunogenic cell death induced by intervening chemotherapy and microenvironment remodeling by fluoropyrimidines, may sensitize ACC to PD-1 blockade. While limited by the lack of archival tissue for translational validation, this hypothesis-generating case warrants further investigation into combination immunotherapy strategies for metastatic ACC.

## 1 Introduction

Adenoid cystic carcinoma (ACC) is a rare malignancy of secretory origin characterized by indolent growth, perineural invasion, and a high propensity for hematogenous metastasis (1). Histologically, ACC can be classified into tubular, cribriform, and solid subtypes. Tumors with a predominantly solid pattern are often diagnosed at a more advanced stage, show a higher tendency for distant metastasis, and are associated with a poorer prognosis (2). Primary intraosseous adenoid cystic carcinoma (PIACC) of the mandible is an extremely rare subset, with 55 cases reported in the English literature to date (3–6). Unlike conventional salivary gland ACC, PIACC arises centrally within the jawbone, often leading to delayed diagnosis and extensive osteolytic destruction (6).

The prognosis for patients with metastatic ACC is generally poor, with a median overall survival of less than 3 years in the metastatic setting (1). Systemic therapy options are limited; cytotoxic chemotherapy yields low objective response rates (<20%), and tyrosine kinase inhibitors (TKIs), despite their disease-stabilizing effects, are often associated with higher-than-expected toxicity that limits long-term administration (7–10). Furthermore, ACC is historically classified as an immunologically “cold” tumor with a low tumor mutational burden (TMB) and minimal immune cell infiltration, resulting in disappointing response rates to immune checkpoint inhibitors (ICIs) in monotherapy trials (11–14).

However, strategies to sensitize ACC to immunotherapy while maintaining long-term tolerability remain largely unexplored. Herein, we present a rare case of *de novo* metastatic mandibular PIACC successfully managed with a multimodal approach, achieving overall survival exceeding 8 years, driven largely by a prolonged response to a PD-1 inhibitor combined with oral fluoropyrimidines.

## 2 Case presentation

### 2.1 Patient information and clinical findings

A 62-year-old male non-smoker presented to the Department of Oral and Maxillofacial Surgery in 2017 with a two-month history of a painless, progressive mass on the left lower gingiva. Physical examination revealed a firm, fixed mass expanding the left mandible without localized sensory or motor deficits.

### 2.2 Diagnostic assessment

**Figure 1:**
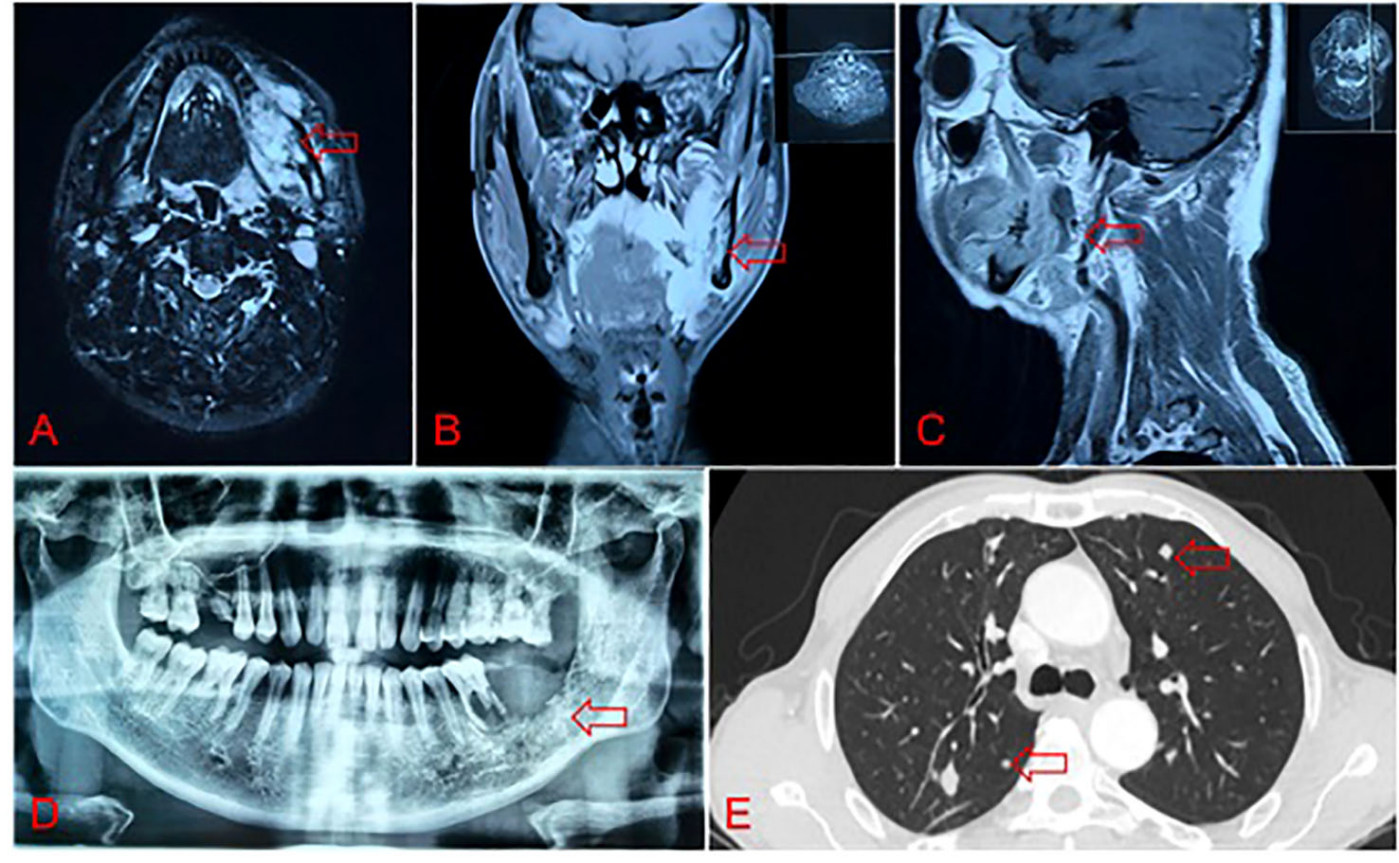
Preoperative imaging of the left mandibular lesion with pulmonary metastases. **(A)** Axial MRI view; **(B)** Coronal MRI view; **(C)** Sagittal MRI view; **(D)** Dental radiograph (periapical X-ray). **(E)** Chest CT scan.

Preoperative computed tomography (CT) and magnetic resonance imaging (MRI) demonstrated an extensive osteolytic lesion in the left mandible with cortical destruction and soft tissue extension (**Figure 1**). Thoracic CT identified multiple bilateral pulmonary nodules, suggestive of synchronous distant metastases.

### 2.3 Therapeutic intervention

#### Locoregional treatment

**Figure 2:**
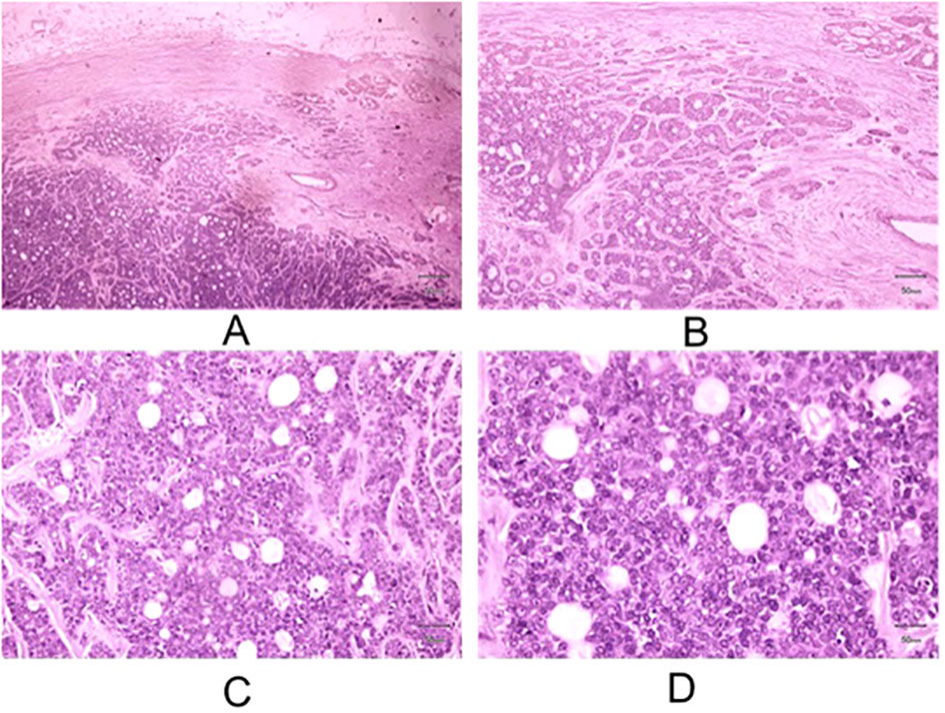
Pathological findings. HE staining: **(A)** HE, ×40: Infiltrative tumor nests within mandibular bone marrow, with hyalinized stroma. **(B)** HE, ×100: Predominant cribriform pattern forming pseudocystic spaces with eosinophilic material and focal tubular areas. **(C)** HE, ×400: Basaloid cells with hyperchromatic nuclei and scant cytoplasm surrounding pseudocysts; no marked atypia. **(D)** HE, ×200: Focal myxomatous and hyalinized stroma, highlighting ductal-myoepithelial biphasic features.

On August 16, 2017, the patient underwent a composite resection of the left mandible, partial maxillectomy, and ipsilateral functional neck dissection. Postoperative histopathology confirmed primary intraosseous adenoid cystic carcinoma (PIACC) of the mandible. Histologically, the tumor predominantly exhibited a cribriform growth pattern with characteristic pseudocystic spaces containing eosinophilic basement membrane–like material, accompanied by focal tubular structures; no predominant solid component was identified (**Figure 2**).

Metastasis was confirmed in 4 of 18 cervical lymph nodes (Levels Ib and IIa/b). The final pathological stage was determined as pT4aN2bM1 (Stage IVC).

**Figure 3:**
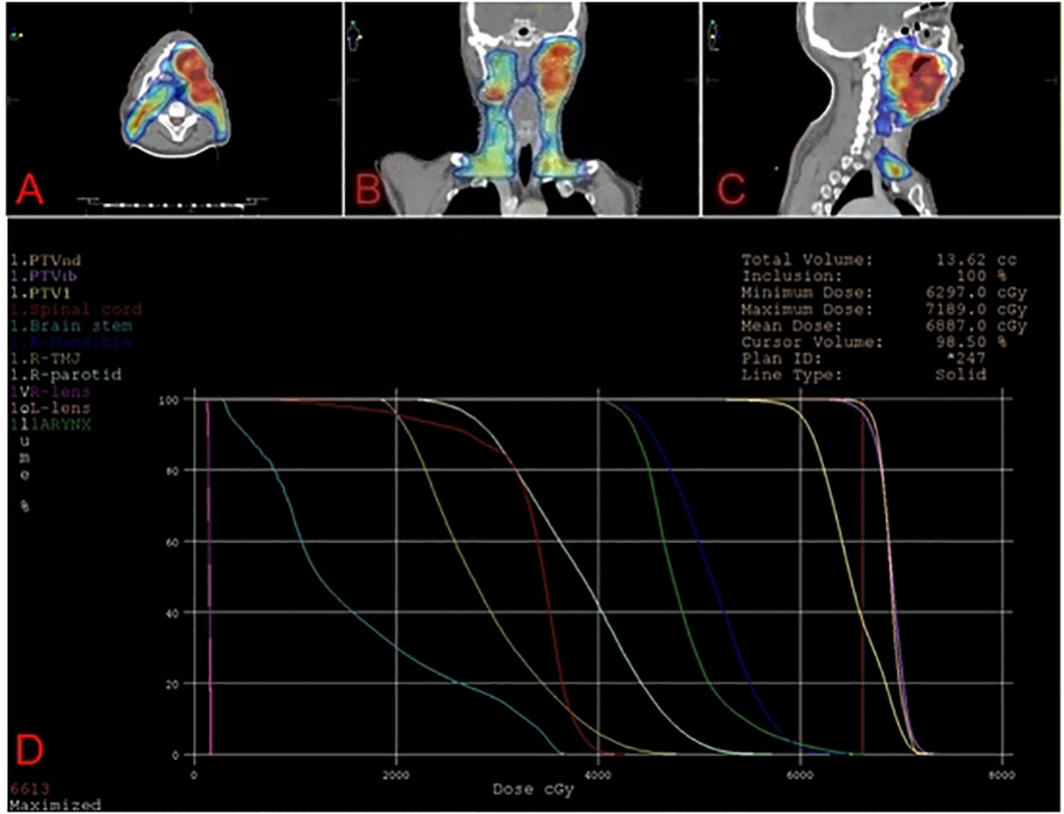
Radiotherapy dose distribution and planning evaluation. **(A-C)** The 95% isodose line encompassing the planned target volumes is displayed on pretreatment planning CT images in the **(A)** axial, **(B)** coronal, and **(C)** sagittal planes. The target volumes are color-coded as follows: tumor bed clinical target volume (PTVtb, purple), nodal clinical target volume (PTVnd, brown), and high-risk clinical target volume (PTV1, yellow). **(D)** The dose-volume histogram.

Given the advanced stage but preserved performance status, aggressive locoregional control was prioritized. Six weeks post-surgery, the patient received adjuvant intensity-modulated radiation therapy (IMRT). A total dose of 66 Gy in 30 fractions was delivered to the tumor bed (PTVtb) and involved nodal levels, while high-risk nodal regions received 54 Gy (**Figure 3**).

#### Systemic management

In December 2017, maintenance therapy with S-1 (tegafur/gimeracil/oteracil, 60 mg BID, days 1–14) was initiated, achieving SD for 36 months. Upon radiologic progression of pulmonary metastases in December 2020, the regimen was escalated to the PD-1 inhibitor sintilimab (200 mg q3w) combined with S-1.

**Figure 4:**
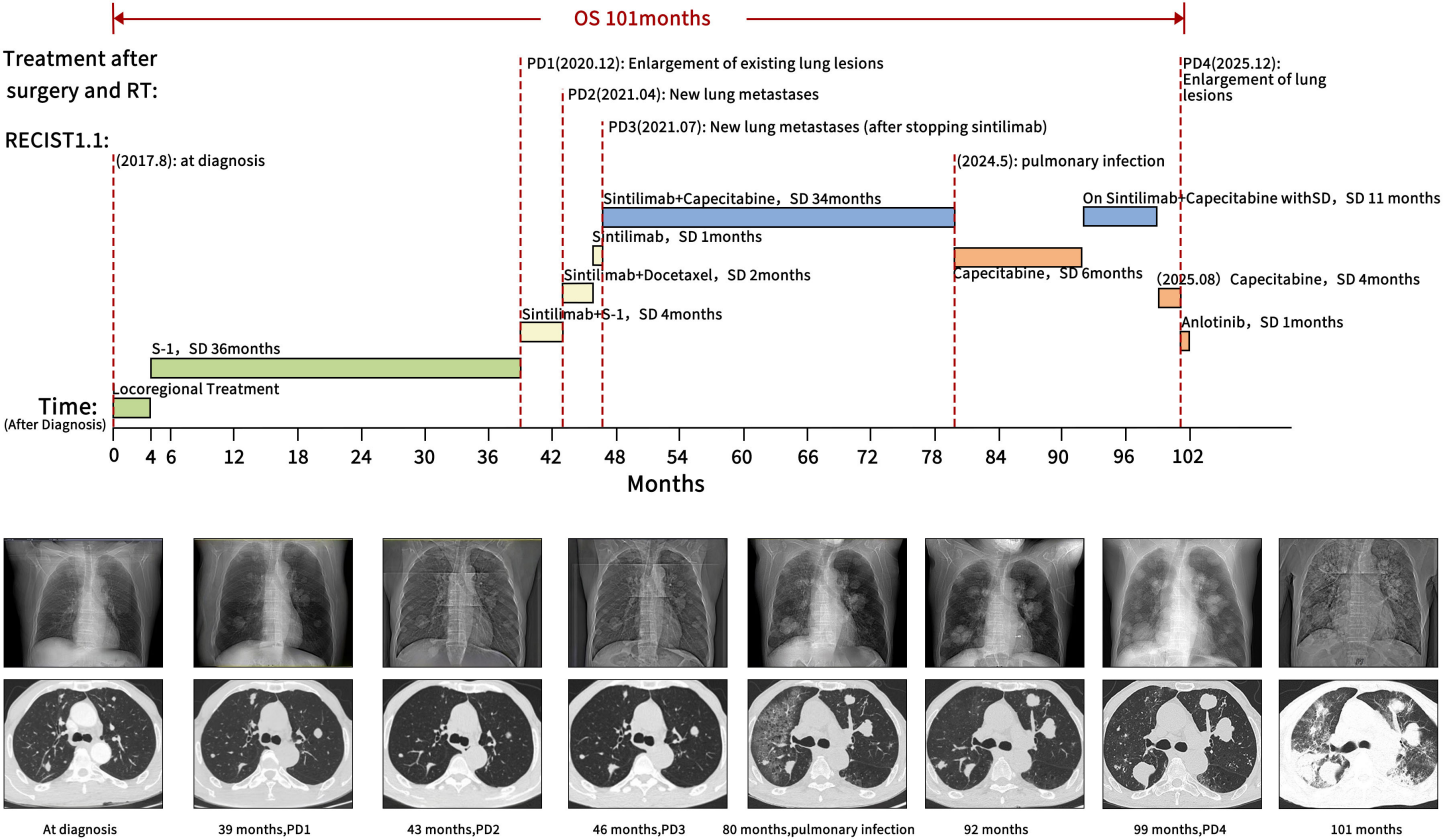
Clinical course and treatment timeline of the patient. The timeline starts at Month 0 (diagnosis in 2017). Key progression events (RECIST 1.1): PD1(2020.12): Enlargement of existing lung lesions; PD2 (2021.04): New lung metastases; PD3(2021.07): New lung metastases (after stopping sintilimab); PD4(2025.12): Progression due to further enlargement of pulmonary lesions (after anlotinib). Total OS: 101 months (death on 22 Jan 2026 due to severe pneumonia).

Following four cycles, the disease showed radiological progression; consequently, the patient was switched to sintilimab plus docetaxel. However, Grade 3 gastrointestinal toxicity necessitated the discontinuation of intravenous chemotherapy after two cycles. Consequently, the treatment was optimized in July 2021 to a combination of sintilimab (200 mg intravenously every 3 weeks) and oral capecitabine (1.5 g BID, days 1–14). This combined immunotherapy regimen was well-tolerated and adopted as long-term maintenance therapy (**Figure 4**).

### 2.4 Follow-up and outcomes

Following the transition to sintilimab and capecitabine in July 2021, the pulmonary metastases exhibited durable stabilization. The patient maintained Stable Disease (SD) for over 4 years on this combination regimen (**Figure 4**).

In August 2025, due to financial constraints, the patient discontinued sintilimab and continued maintenance therapy with oral capecitabine monotherapy. Unfortunately, follow-up imaging in December 2025 revealed disease progression (PD), suggesting that the prolonged disease control was likely driven by the synergy between the PD-1 inhibitor and the fluoropyrimidine.

Subsequently, the patient was switched to salvage targeted therapy with anlotinib (12 mg, once daily, days 1–14). After two cycles, radiological evaluation indicated regained tumor stability (SD). However, the patient subsequently developed severe pneumonia (presumed community-acquired, though late-onset treatment-related pulmonary toxicity could not be entirely excluded) and passed away on January 22, 2026. The total overall survival (OS) from the initial diagnosis was approximately 101 months (8.4 years).

## 3 Discussion

Primary intraosseous adenoid cystic carcinoma (PIACC) of the mandible is an exceptionally rare malignancy. Unlike conventional salivary gland ACC, PIACC arises within the jawbone, often leading to delayed diagnosis and extensive osteolytic destruction. To date, 55 cases have been documented in the literature (3–6). This case illustrates a distinct survival advantage in a patient with *de novo* metastatic PIACC, achieved through aggressive locoregional control followed by a novel chemo-immunotherapy maintenance strategy.

### 3.1 Diagnostic certainty and local control

The diagnosis of PIACC is often challenging. In this case, the diagnosis was established strictly based on the intraosseous epicenter of the lesion, characteristic cribriform histology, and the absence of any primary lesion in the adjacent major salivary glands or sinonasal tract (15).

The management of metastatic ACC remains challenging. While distant metastases (M1) typically preclude curative surgery in many solid tumors, ACC is characterized by a paradoxical “indolent but relentless” progression. Retrospective data suggest that resection of the primary tumor significantly improves overall survival (OS) in metastatic head and neck ACC by preventing fatal locoregional complications and reducing the total tumor burden (16). In our case, the patient underwent composite resection and adjuvant radiotherapy, achieving locoregional control for over 8 years. This experience supports the concept that durable locoregional control may serve as a prerequisite for long-term survival in metastatic ACC, preserving quality of life and creating a therapeutic window for systemic immune-modulating strategies.

### 3.2 Efficacy of immunotherapy in ACC: a paradigm shift

Historically, ACC is classified as an immunologically “cold” tumor, characterized by a low tumor mutational burden (TMB) and an immune-excluded microenvironment, frequently associated with oncogenic drivers such as the MYB-NFIB fusion gene (17). Consequently, clinical trials evaluating immune checkpoint inhibitors (ICIs) as monotherapy have shown limited efficacy. For instance, the KEYNOTE-028 study (pembrolizumab) reported an objective response rate (ORR) of only 12% (14), and a phase II trial of nivolumab demonstrated an ORR of 8.7% (13). Therefore, the sustained control observed in our case was unlikely to be solely attributable to the PD-1 inhibitor alone. However, recent evidence suggests that combinatorial strategies may overcome this intrinsic immune resistance. Notably, a recent phase II study evaluating the combination of nivolumab and ipilimumab for metastatic/recurrent ACC of all anatomic sites demonstrated a relatively favorable disease control rate (18). This pivotal finding indicates a paradigm shift: while ACC may be refractory to PD-1 blockade alone, combination immunotherapy regimens can effectively modulate the tumor microenvironment (TME) to achieve meaningful clinical benefit. The sustained, durable stabilization (>47 months) observed in our case using a metronomic combination of sintilimab and oral capecitabine strongly aligns with this emerging concept of combinatorial synergy.

### 3.3 Putative synergistic mechanisms of the immuno-metronomic regimen

An intriguing aspect of this patient’s clinical course is the differential response to systemic therapy: the initial sintilimab plus S-1 regimen failed, whereas the subsequent sintilimab plus capecitabine combination (preceded by docetaxel) yielded a durable response. We hypothesize that this phenomenon was driven by several overlapping mechanisms:

Depletion of Suppressive Cells: Although both S-1 and capecitabine are fluoropyrimidines, their pharmacokinetic profiles and tissue distributions differ significantly. Capecitabine requires a three-step enzymatic conversion involving thymidine phosphorylase (TP), which may be upregulated in the TME following previous treatments. Oral fluoropyrimidines selectively deplete myeloid-derived suppressor cells (MDSCs) and regulatory T cells (Tregs), which are metabolically more sensitive to these agents than cytotoxic effector T cells. This reduction in immunosuppression helps “release the brakes” within the tumor microenvironment (19–22).

Immunogenic Cell Death (ICD): Chemotherapy can induce ICD, promoting the release of tumor-associated antigens and damage-associated molecular patterns (DAMPs), thereby enhancing antigen presentation by dendritic cells (23, 24). The brief intervening exposure to docetaxel prior to capecitabine maintenance may have acted as a powerful immunological trigger.

Cryptic Immunogenic Profiles: It is highly plausible that this specific tumor harbored an unusual molecular profile (e.g., elevated TMB, frameshift mutations, or a lack of the classic MYB fusion) that conferred intrinsic sensitivity to immunotherapy. Consistent with this hypothesis, a recent anecdotal case report in the literature detailed a complete response to dual immunotherapy in a lacrimal gland ACC despite a lack of conventional immunotherapy biomarkers (25). This suggests that a subset of ACCs possesses a favorable molecular profile capable of driving robust immune responses. Notably, disease progression occurred rapidly after the discontinuation of the PD-1 inhibitor in August 2025, while the patient was on capecitabine monotherapy. This clinical observation strongly reinforces our hypothesis that the therapeutic benefit was driven by the synergism between the checkpoint inhibitor and the fluoropyrimidine, rather than by chemotherapy alone.

### 3.4 Safety and comparison with targeted therapies

Following disease progression upon the discontinuation of immunotherapy, anlotinib was selected as a salvage therapy. As a multi-target tyrosine kinase inhibitor (TKI) blocking VEGF, c-Kit, and FGFR pathways, anlotinib targets the angiogenic dependencies of ACC. The regained disease stabilization observed in our case corroborates emerging clinical data; a recent retrospective study reported a disease control rate (DCR) of 63.2% for anlotinib in metastatic ACC (26). This clinical course underscores the value of VEGFR-targeted TKIs as effective rescue interventions when immuno-metronomic strategies are exhausted.

While multi-kinase inhibitors (TKIs) like lenvatinib have shown higher response rates in ACC (ORR ~16%, SD ~75%), they are frequently associated with substantial Grade 3–4 toxicities (e.g., hypertension, proteinuria) that limit long-term adherence (8, 27). In contrast, the “immuno-metronomic” regimen of sintilimab plus capecitabine in our case demonstrated an excellent safety profile, allowing for uninterrupted treatment for nearly four years with only manageable Grade 1 hand-foot syndrome. However, the clinical course was ultimately complicated by a fatal severe pneumonia. While presumed to be community-acquired, delayed immune-mediated pneumonitis from prolonged sintilimab exposure or TKI-related pulmonary toxicity from anlotinib cannot be definitively ruled out. Consequently, the brief “stable disease” designation achieved after only two cycles of anlotinib must be interpreted with great caution.

### 3.5 Limitations

This study is limited by its nature as a single case report. The lack of comprehensive molecular profiling (e.g., MYB status, TMB, and PD-L1 expression) limits our ability to identify specific biomarkers of response. Despite our rigorous efforts, supplementary immunohistochemistry was precluded by insurmountable medico-legal barriers in retrieving the 2017 archival tissues following the patient’s death, compounded by the high risk of severe antigen degradation over nearly a decade. Additionally, while the radiological diagnosis of pulmonary metastases was typical for ACC, biopsy confirmation of the lung lesions was not obtained, aligning with standard practice to minimize invasiveness. Despite these limitations, the exceptional durability of disease control observed in this case provides a unique clinical window into potential immune-modulating mechanisms and supports further exploration of immuno-metronomic strategies in immunologically cold tumors.

## 4 Conclusion

This case demonstrates that *de novo* metastatic PIACC may be amenable to long-term disease control. Aggressive locoregional treatment provided a critical foundation for survival, while immuno-metronomic maintenance therapy combining PD-1 blockade with oral capecitabine was associated with exceptional durability and manageable tolerability. Although limited by the absence of molecular profiling, these findings align with emerging data supporting combination immunotherapy for ACC, warranting further prospective investigation as a promising strategy to overcome immune resistance in immunologically cold tumors.

## Data Availability

The original contributions presented in the study are included in the article/supplementary material, further inquiries can be directed to the corresponding author/s.
